# Development and Validation of a DeepSurv Nomogram to Predict Survival Outcomes and Guide Personalized Adjuvant Chemotherapy in Non-Small Cell Lung Cancer

**DOI:** 10.3389/fonc.2022.895014

**Published:** 2022-06-23

**Authors:** Bin Yang, Chengxing Liu, Ren Wu, Jing Zhong, Ang Li, Lu Ma, Jian Zhong, Saisai Yin, Changsheng Zhou, Yingqian Ge, Xinwei Tao, Longjiang Zhang, Guangming Lu

**Affiliations:** ^1^Medical Imaging Center, Calmette Hospital and The First Hospital of Kunming (Affiliated Calmette Hospital of Kunming Medical University), Kunming, China; ^2^Department of Medical Imaging, Affiliated Jinling Hospital, Medical School of Nanjing University, Nanjing, China; ^3^Department of Cardiology, Tongji Hospital, Tongji University School of Medicine, Shanghai, China; ^4^Department of Medical Imaging, Jinling Hospital, Nanjing Medical University, Nanjing, China; ^5^Siemens Healthineers Ltd., Shanghai, China

**Keywords:** chemotherapy, DeepSurv, non-small cell lung cancer, survival outcome, radiomics

## Abstract

**Objective:**

To develop and validate a DeepSurv nomogram based on radiomic features extracted from computed tomography images and clinicopathological factors, to predict the overall survival and guide individualized adjuvant chemotherapy in patients with non-small cell lung cancer (NSCLC).

**Patients and Methods:**

This retrospective study involved 976 consecutive patients with NSCLC (training cohort, n=683; validation cohort, n=293). DeepSurv was constructed based on 1,227 radiomic features, and the risk score was calculated for each patient as the output. A clinical multivariate Cox regression model was built with clinicopathological factors to determine the independent risk factors. Finally, a DeepSurv nomogram was constructed by integrating the risk score and independent clinicopathological factors. The discrimination capability, calibration, and clinical usefulness of the nomogram performance were assessed using concordance index evaluation, the Greenwood-Nam-D’Agostino test, and decision curve analysis, respectively. The treatment strategy was analyzed using a Kaplan–Meier curve and log-rank test for the high- and low-risk groups.

**Results:**

The DeepSurv nomogram yielded a significantly better concordance index (training cohort, 0.821; validation cohort 0.768) with goodness-of-fit (*P*<0.05). The risk score, age, thyroid transcription factor-1, Ki-67, and disease stage were the independent risk factors for NSCLC.The Greenwood-Nam-D’Agostino test showed good calibration performance (*P*=0.39). Both high- and low-risk patients did not benefit from adjuvant chemotherapy, and chemotherapy in low-risk groups may lead to a poorer prognosis.

**Conclusions:**

The DeepSurv nomogram, which is based on the risk score and independent risk factors, had good predictive performance for survival outcome. Further, it could be used to guide personalized adjuvant chemotherapy in patients with NSCLC.

## Introduction

Lung cancer is associated with the highest morbidity and mortality rates globally. Approximately 80–85% of lung cancers are non-small cell lung cancers (NSCLCs) (1–3). There are several clinicopathologic factors and systems to predict prognosis; however, each has its limitations. The tumor-node-metastasis (TNM) staging system is an important prognostic method for early lung cancer after surgery (4–6); however, patients with the same TNM stage may have completely different prognoses, indicating that pathological staging alone is not an ideal tool for prognosis (7–9). Some studies believe that traditional clinicopathological factors, including age, sex, pathological type, and tumor grade, are related to the prognosis of NSCLC (10). Owing to developments in biological gene technology, the biological and genetic characteristics related to survival can be included, thus greatly improving the assessment of prognosis. Although some genes related to lung cancer have been successfully used in clinical settings, there are associated ethical and clinical limitations. These invasive methods cannot fully reflect the spatiotemporal heterogeneity of tumors (11–14), which is closely related to cell proliferation, necrosis, hypoxia, and angiogenesis (15, 16). Therefore, a new prognosis evaluation method is required for prognosis, identifying patients with a high-risk of recurrence, and recommending individualized therapy (17).

Artificial intelligence is an emerging field in oncology with promising results for prognosis and monitoring the treatment response (18–20). Radiomics as a method of quantitative machine learning that can quantify the temporal and spatial heterogeneity of tumor tissue, and provide guidance for precise personalized diagnosis and treatment (21). Some studies have attempted to improve the prediction performance of various cancers using computed tomography (CT) or positron emission tomography(PET)/CT radiomic technology. Studies have found that radiomics combined with traditional staging systems and other clinicopathological factors may improve the prediction of tumor prognosis (22–24). However, the prediction of the model is generally inaccurate owing to the small sample size (25–27). DeepSurv (28), proposed in 2018, is a multi-layer feed-forward network with a negative log partial likelihood output parameterized by the weights of the network. DeepSurv is composed of an Artificial Neural Network (ANN) model and Cox proportional hazards (CPH) model. The former is used as the front-end model to select features, while the latter uses the feature variables obtained from the regression of the neural network model as the input to calculate the risk model (29, 30). Hence, we developed a DeepSurv nomogram based on radiomic features to improve risk stratification capability and discrimination with a more accurate prediction of prognosis. However, there is still no feasible unified standard in clinical practice for how to judge the risk of recurrence from an overall perspective to achieve individualized treatment. National Comprehensive Cancer Network guidelines pointed out that adjuvant chemotherapy can be considered for patients with high risk factors of early NSCLC after surgery; however, whether postoperative adjuvant chemotherapy is needed in stages NSCLC IB and IIA remains controversial. Owing to the lack of individualized treatment options, patients who cannot benefit from adjuvant chemotherapy suffer from the toxic damage and economic loss of chemotherapy (31). Therefore, identifying patients who would benefit from adjuvant chemotherapy is key to individualized treatment.

Therefore, this study aimed to construct a DeepSurv nomogram to predict the prognosis of NSCLC based on CT radiomic features and independent risk factors, and to conduct risk stratification to guide individualized adjuvant chemotherapy after early lung cancer surgery.

## Materials and Methods

### Patients and Clinicopathological Data

**Figure 1:**
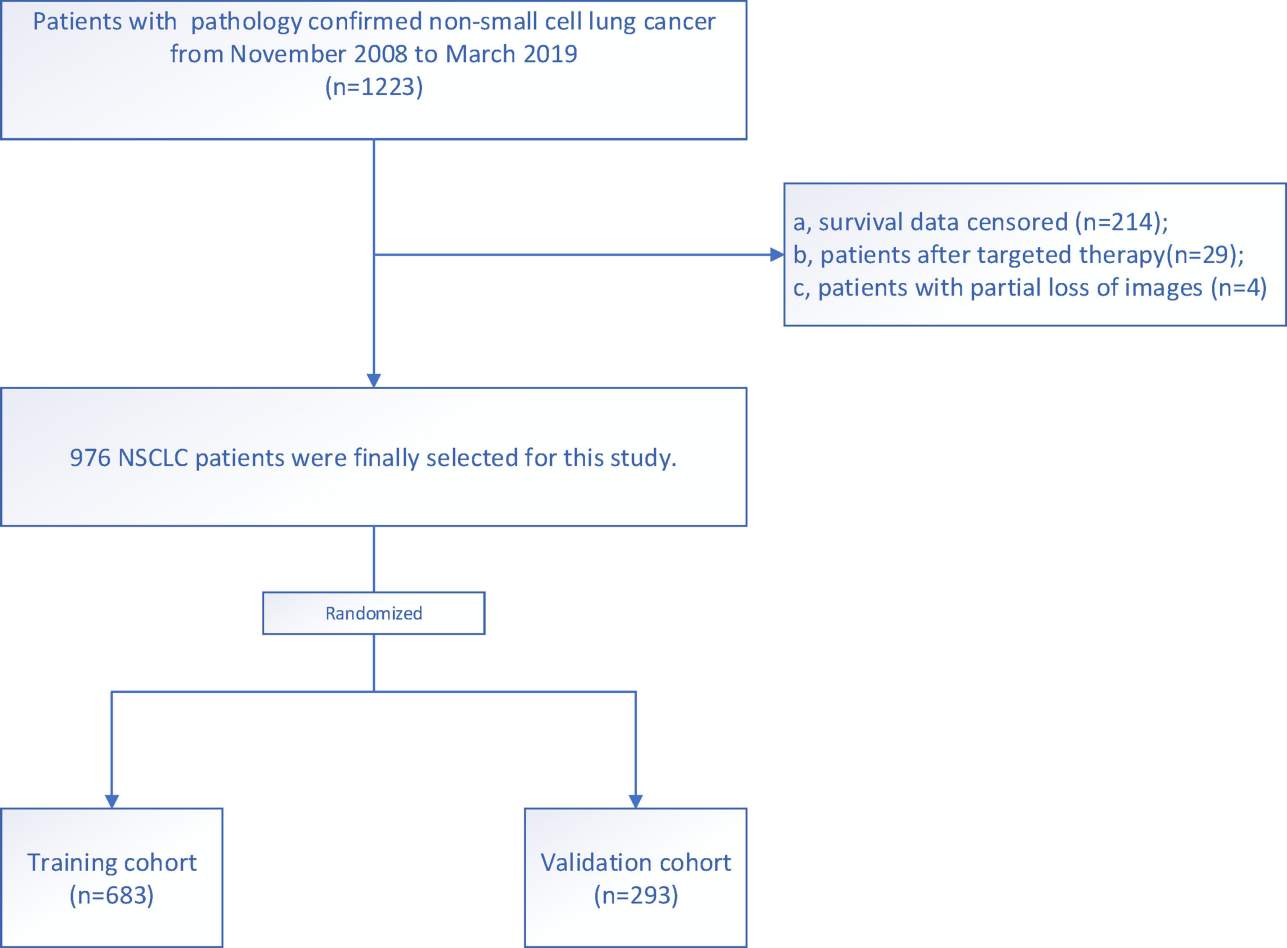
Flow diagram of the enrollment of patients with non-small cell lung cancer.

The institutional ethics review board of the affiliated Jinling Hospital, Medical School of Nanjing University, approved this retrospective study and waived the need to obtain informed consent. The institutional database was searched for medical records from November 2008 to March 2019 to identify patients with histologically confirmed NSCLC (stages IA, IB, IIA, IIB, and IIIA). The inclusion criteria were as follows: a) patients with NSCLC who underwent CT before treatment between November 2008 and March 2019; b) patients diagnosed with stage IA, IB, IIA, IIB, or IIIA NSCLC, as confirmed by histopathological examination according to the American Joint Committee on Cancer eighth edition TNM classification and staging system; and c) patients with complete imaging and clinicopathological data. The exclusion criteria were as follows: a) patients with censored survival data (n=214); b) patients who had undergone targeted therapy (n=29); and c) patients with partial loss of images (n=4). Finally, 976 patients were included. We randomly divided the patients into: a) training cohort (n=683) and b) validation cohort (n=293) using a 7:3 ratio (**Figure 1**). For each patient, we collected the baseline clinicopathological characteristics, including age, sex, smoking status, family history, histologic subtype, stage (T stage, N stage, and clinical stage), chemotherapy, C-reactive protein (mg/L), thyroid transcription factor-1 (TTF-1), and Ki-67, from the medical records. The survival information of these patients was obtained through telephone calls. Follow-up data were collected from November 2008 to March 2020. The endpoint of this study was the overall survival (OS), which is defined as the period from the date of CT examination to the date of telephone follow-up or patient’s death.

### Image Acquisition and Reconstruction Parameters

All patients underwent unenhanced CT imaging of the lungs with one of three multi-detector row CT systems (SOMATOM definition flash, Siemens Healthineers, Erlangen, Germany; SOMATOM Emotion, Siemens AG, Erlangen, Germany; and SOMATOM Perspective). Patients were placed in the supine position with both hands raised, and any metallic foreign bodies were removed from the chest. The scanning range was from the thoracic entrance to the underlying layer of the lung, with a single breath-hold scan at the end of inspiration, using the spiral scanning mode. The CT parameters were as follows: 120 kVp for SOMATOM definition flash or 130 kVp for SOMATOM emotion and perspective; reference mAs, 160 mAs; reconstructed with 1 mm or 1.25 mm slice thickness by a standard lung kernel. These CT images were retrieved from the picture archiving and communication system.

### Image Segmentation

A volume of interest(VOI) was drawn semi-automatically around the tumor by a chest radiologist (Y.B., 9 years of experience) and confirmed by another chest radiologist (Z.J., 15 years of experience). Both the radiologists were blinded to the patients’ clinical information. First, we imported the CT images into the radiomics prototype software. Next, the doctor drew a line across the tumor’s boundary, the tool automatically found the neighboring voxels in 3-D space with the same gray-level using an algorithm, a random walker-based lesion segmentation for solid and subsolid lung lesions (32). If the segmentation was not satisfactory, the operators corrected it manually in the 3-D domain using the radiomics prototype. To test intra-class reproducibility, 100 cases were randomly selected and segmented twice by one radiologist (Y.B., 9 years of experience). To test inter-class reproducibility, all 100 cases were segmented by two radiologists (Y.B. and Z.J.). Spearman’s correlation analyses were used to test the reproducibility of the features. Features with Rho>0.8 were selected for further analysis.

### Radiomic Feature Extraction and DeepSurv Model Construction

**Figure 2:**
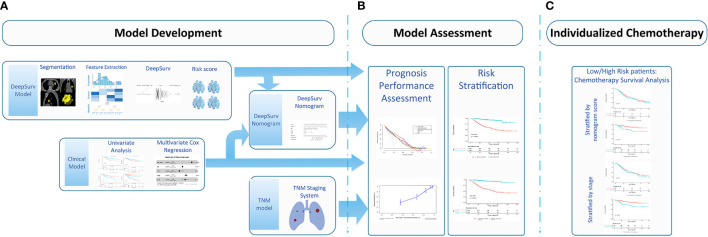
Workflow for developing the DeepSurv nomograma. **(A)** Computed tomography (CT) image segmentation was performed using semiautomatic segmentation on the radiomics prototype software (Radiomics, Frontier, Siemens); **(B)** The radiomic features from the volumes of interest were then computed with CT images on a prototype, including first-order statistics, shape, size, and texture features; **(C)** DeepSurv was developed based on the radiomic features. The risk score was calculated for each patient, and the patients were stratified into high- and low- risk groups according to the median risk score; DeepSurv nomogram for 3 and 5-year overall survival (OS) was generated for non-small cell lung cancer (NSCLC) patients. Calibration curves are drawn for the DeepSurv nomogram-predicted and actual survival of patients. The risk stratification could be used to guide individualized adjuvant chemotherapy for high-risk patients.

Our study adhered to the Image Biomarker Standardization Initiative (IBSI) guidelines (33). The software syngo *via* Frontier 1.2.1 (version VB10B, Siemens Healthineers, Germany) was IBSI-compliant. The medical image series were resampled to the 1 mm ×1 mm × 1 mm voxel size before subsequent feature extraction steps. B-spline interpolation was used for resampling. The bin width size was set at 25 when creating a histogram for the discretization of the gray image levels. After preprocessing, the extracted radiomic feature groups based on original images were as follows: 18 first-order features; 17 size and shape features; 75 texture encoding features, including 14 gray-level dependence matrix features, 24 gray-level co-occurrence matrix features, 16 gray-level run-length matrix features, 16 gray-level size zone matrix features, and five neighboring gray-tone difference matrix features. Laplacian of Gaussian filtering, wavelet filtering, nonlinear intensity transformations (including square, square root, logarithm, and exponential operations), and wavelet-transformed images (including three directions [x, y, z]; LLL, LLH, LHL, LHH, HLL, HLH, HHL, and HHH) were also generated. In total, 1,227 radiomic features were extracted from each lesion. A deep learning model based on the radiomic features was composed of ANN and CPH models. ANN was used as a preposition model to filter the features of the samples, and the CPH was used to calculate the risk function by connecting the ANN model with the neural network regression model. ANN was composed of one output and four hidden layers. The activation function used by the hidden layer is the scaled exponential linear unit. The hidden layer also included a dropout layer to improve the generalization of the model, in addition to the fully connected neural network layer. The Adam optimizer used the negative log partial likelihood as the loss function, combined with batch normalization, weight decay regularization, and learning rate scheduling for training (learning rate decay rate, 3.173e-4; dropout ratio, 0.401). The final output of ANN was the covariate in the Cox model to return all the covariate features in the original sample to a feature or θ(x) in the formula. The basic risk model used in the Cox model was obtained by the Nelson–Aalen model, which is a linear univariate risk model that takes the event and time as the input function b_0 (t). A DeepSurv model was established based on the radiomic features. The output was the risk score for each patient **(** **Figure 2****)**.

### Clinical Model Development

The clinicopathological factors were analyzed using a univariate CPH regression analysis. The predictors with *P*<0.05 were included in the multivariate CPH regression analysis to identify the independent risk factors. The final model was selected by backward stepwise elimination, with Akaike information criteria as the stopping rule (34).

### Clinical Plus DeepSurv Model Development and DeepSurv Nomogram Construction

The multimodal features and parameters, including the risk score and independent clinicopathological factors, were integrated into a single predictive model based on the multivariate CPH model. Based on the multivariable CPH regression analysis in the training cohort, a DeepSurv nomogram was developed. Thereafter, a DeepSurv nomogram risk score was derived for each patient.

### Guide to Individualized Adjuvant Chemotherapy for Patients With NSCLC

The patients were divided into high- and low- risk groups according to the DeepSurv nomogram score. The treatment strategy was explored separately in high- and low-risk cohorts using a Kaplan–Meier analysis and log-rank test to find the cohort that benefited from chemotherapy. According to the lung cancer diagnosis and treatment recommendations, adjuvant chemotherapy was not recommended for NSCLC stages IA, IB (including lung cancer with high-risk factors), and IIA after complete resection, owing to the lack of high-level evidence (35–37). Therefore, we divided patients into low-risk (IA, IB, and IIA) and high-risk groups (IIB and IIIA), and conducted a Kaplan–Meier analysis and log-rank test on the survival rates of patients treated with adjuvant chemotherapy, to evaluate whether patients would benefit from the therapy.

### Statistical Analysis

The differences in age, sex, TNM stage, and survival time for the training and validation datasets were assessed using the Mann–Whitney U test for continuous variables and the χ^2^ test for categorized variables. Model discrimination was measured using the concordance index (C-index) and compared for the two datasets. The proportional hazards assumption of the models was verified by examining the scaled Schoenfeld residual plots. Survival curves were generated using the Kaplan–Meier method and compared by two-sided log-rank tests. Calibration was evaluated for 3 and 5 years using a calibration plot, a graphical representation of the relationship between the observed and predicted survival, and the Greenwood-Nam-D’Agostino (GND) goodness-of-fit test (38). The prediction error of models was assessed using the “Boot632plus” split method with 1,000 iterations, to calculate estimates of prediction error curves. These estimates were summarized as the integrated Brier score, which represents a valid measure of overall model performance. This could range from 0, for a perfect model, to 0.25, for a noninformative model with a 50% incidence of the outcome (39). R software (version 3.4.4, http://www. r-project.org) was used for the statistical analysis. The DeepSurv model was developed using Python (Python package [lifeline 0.24.3, Python version 3.7]). The optimal cutoff point for continuous prognostic markers in the survival analysis was determined using X-tile (version 3.6.1; Yale University School of Medicine, New Haven, Conn) (40). The proportional hazards assumption of the models was verified using scaled Schoenfeld residual plots. The details of the packages used are described in the Appendix. All the codes are available: https://github.com/tomato08217/DeepSurvLungCa. All statistical tests were two-sided, with a significance level of 0.05.

## Results

### Clinicopathological Characteristics

The patients in our study were divided into two groups: the training cohort with 683 patients (378 males and 305 females; median age, 62 years; range, 54 to 69 years) and the validation cohort with 293 patients (155 males and 138 females; median age, 62 years; range, 56 to 68 years). The clinicopathological characteristics of all patients in the training and validation cohorts are listed in **Table 1**.

**Table 1:** Demographic and clinicopathologic characteristics of patients in the training and validation cohorts.

Demographic or clinicopathologic characteristics	Training cohort (n = 683)	Validation cohort (n = 293)	*p*-value
Age (median [IQR])	62.00 [54.00, 68.00]	63.00 [55.00,68.00]	0.893
Sex, n (%) Male Female	378.00 (55.30) 305.00 (44.70)	155.00 (52.90) 138.00 (47.10)	0.527
Smoking status ,n (%) No	412.00 (60.30)	180.00 (61.40)	0.799
Yes	271.00 (39.70)	113.00 (38.60)	
Family history, n (%) No	654.00 (95.80)	283.00 (96.60)	0.667
Yes	29.00 ( 4.20)	10.00 (3.40)	
TTF-1,n (%)			0.849
Negative Positive	428.00 (62.70) 255.00 (37.30)	181.00 (61.80) 112.00 (38.20)	
Ki-67,n (%) Low expression High expression	518.00 (75.80) 165.00 (24.20)	234.00 (79.90) 59.00 (20.10)	0.198
Histologic type,n (%) Adenocarcinoma	525.00 (76.90)	219.00 (74.74)	0.541
Squamous cell carcinoma	142.00 (20.80)	69.00 (23.55)	
NOS	16.00 (2.30)	5.00 (1.71)	
Stage, n (%) I A	360.00 (52.71)	164.00 (56.00)	0.286
I B	77.00 (11.27)	20.00 (6.80)	
II A	31.00 (4.54)	16.00 (5.50)	
II B	85.00 (12.45)	39.00 (13.30)	
III A	130.00 (19.03)	54.00 (18.40)	
T stage , n (%) T1	425.00 (62.23)	195.00 (66.55)	0.534
T2	171.00 (25.03)	67.00 (22.87)	
T3	59.00 (8.64)	19.00 (6.48)	
T4	28.00 (4.10)	12.00 (4.10)	
N stage,n (%) N0 N1 N2	523.00 (76.57) 73.00 (10.69) 87.00 (12.74)	222.00 (75.77) 33.00 (11.26) 38.00 (12.97)	0.957
Chemotherapy (%) No Yes	515.00 (75.40) 168.00 (24.60)	220.00 (75.10) 73.00 (24.90)	0.981
Survival time (median [IQR])	30.20 [18.50, 52.62]	29.50 [18.85,52.10]	0.905
PLT (median [IQR])	207.00 [169.00, 248.00]	201.00 [165.00,249.00]	0.700
Neutrophil (median [IQR])	61.50 [54.90, 67.80]	59.80 [53.70, 66.00]	0.026
Monocytes (median [IQR])	6.00 [5.15, 7.50]	6.50 [5.30, 7.70]	0.113
lymphocyte (median [IQR])	29.00 [22.95, 35.95]	30.40 [24.00, 37.00]	0.136
CPR (median [IQR])	1.30 [0.50, 3.90]	1.10 [0.50, 4.20]	0.895
NLR (median [IQR])	2.10 [1.50, 3.00]	1.90 [1.50, 2.80]	0.090
LMR (median [IQR])	4.60 [3.40, 6.25]	4.50 [3.50, 6.30]	0.977

### Analysis of Clinicopathological Factors and Construction of Clinical Model

**Figure 3:**
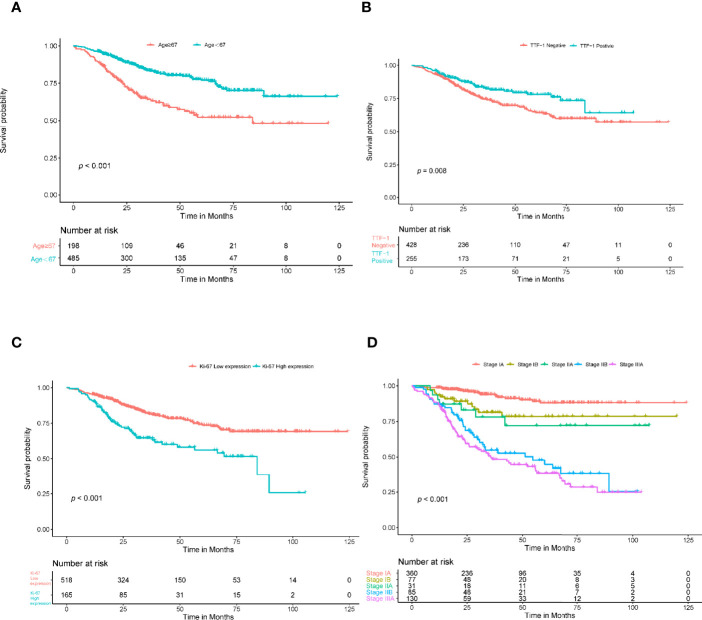
Kaplan-Meier survival analysis for age, TTF-1, Ki-67, and stage. The patients were stratified into high- and low-risk groups based on age **(A)** (*P*<0.001, log-rank test), TTF-1 **(B)** (*P*<0.008, log-rank test), Ki-67 **(C)** (*P*<0.001, log-rank test), and stage **(D)** (*P*<0.001, log-rank test).

In the univariate Cox regression, age, sex, smoking, thyroid transcription factor-1(TTF-1), Ki-67, histologic type, and stage showed significant predictive values (*P*<0.05). After integrating these factors into the multivariate CPH model and using the Akaike information criteria(AIC)as a backward stepwise stopping rule, only the following variables emerged as predictive: age (older than 67 years) (HR, 1.039; 95% CI, 1.021–1.058), TTF-1 (positive) (HR, 0.623; 95% CI, 0.438–0.885), Ki-67 (high expression) (HR, 1.663; 95% CI, 1.191–2.322), stage IB (HR, 2.057; 95% CI, 1.043–4.057), stage IIA (HR, 2.431; 95% CI, 1.038–5.693), stage IIB (HR, 6.875; 95% CI, 4.110–11.503), and stage IIIA (HR, 8.731; 95% CI, 5.474–13.927). A log-rank test was performed, and Kaplan–Meier curves were plotted to test the risk stratification ability. **Figures 3A–D** shows the survival probability of the patients in the high- and low-risk cohorts. The results of the log-rank test indicated significant discrimination between the groups. The C-index values of the clinical model constructed by age, TTF-1, Ki-67, and disease stage were 0.788 and 0.739 in the training and validation cohorts, respectively. The TNM model in **Table 2** was established based on multivariate Cox regression analysis, and the C-index of training and validation cohorts were 0.749 and 0.745, respectively.

**Table 2:** Multivariate Cox proportional hazards (CPH) model regression analysis of independent risk factors.

Independent risk factors	Clinical model					DeepSurv model			
	HR	*P* value	95% CI			HR	*P* value	95% CI	
			Lower	Upper				Lower	Upper
Risk score						2.382	<0.001	1.799	3.155
Age	1.039	<0.001	1.021	1.058		1.030	0.001	1.011	1.048
Ki-67	1.663	0.002	1.191	2.322		1.280	0.155	0.911	1.800
TTF-1	0.623	0.008	0.438	0.885		0.782	0.177	0.546	1.118
IB stage	2.057	0.040	1.043	4.057		1.168	0.600	0.582	2.346
IIA stage	2.431	0.060	1.038	5.693		1.294	0.500	0.543	3.082
IIB stage	6.875	<0.001	4.110	11.503		3.039	<0.001	1.730	5.335
IIIA stage	8.731	<0.001	5.474	13.927		3.578	<0.001	2.107	6.076

HR, hazard ratio; TTF-1, thyroid transcription factor-1.

### Construction and Assessment of the Multimodality Prediction Model

**Figure 4:**
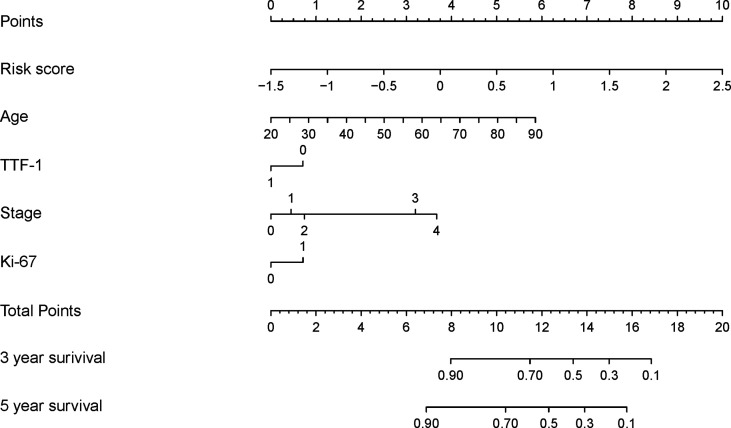
Development of the DeepSurv nomogram by integrating the risk score combined with the clinicopathological factors in the training cohort.

**Figure 5:**
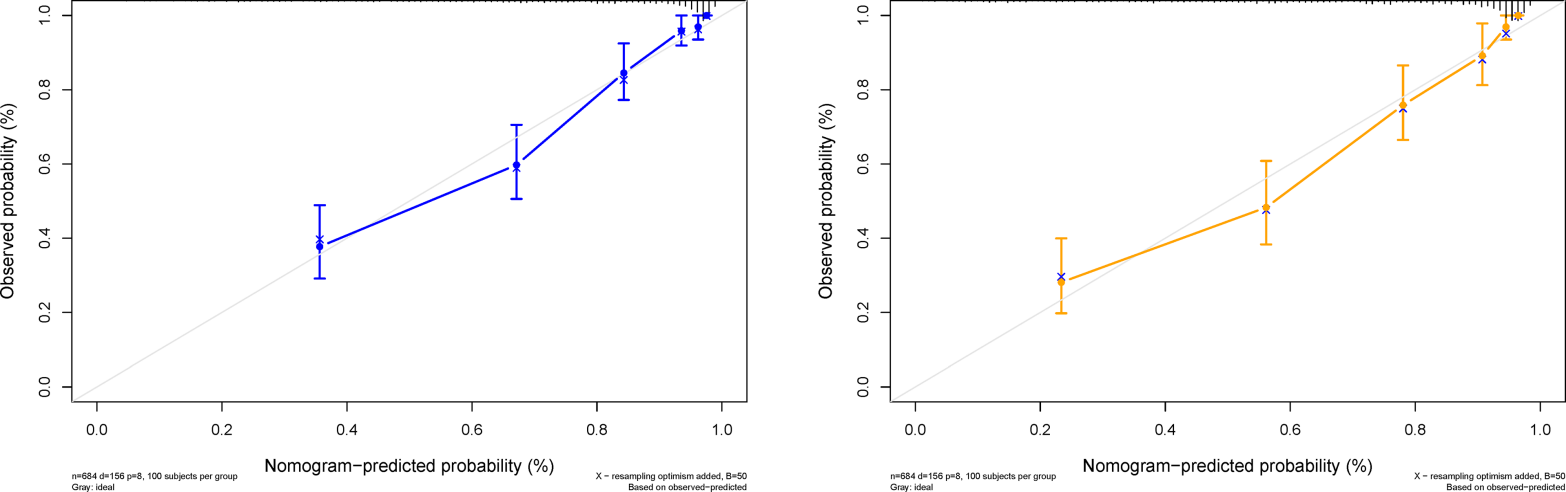
Predicted probability of the 3- and 5-year survival time. The calibration curve of the DeepSurv nomogram. The diagonal gray line represents an ideal evaluation, and the solid blue and yellow lines represent the performance of the DeepSurv nomogram.

**Figure 6:**
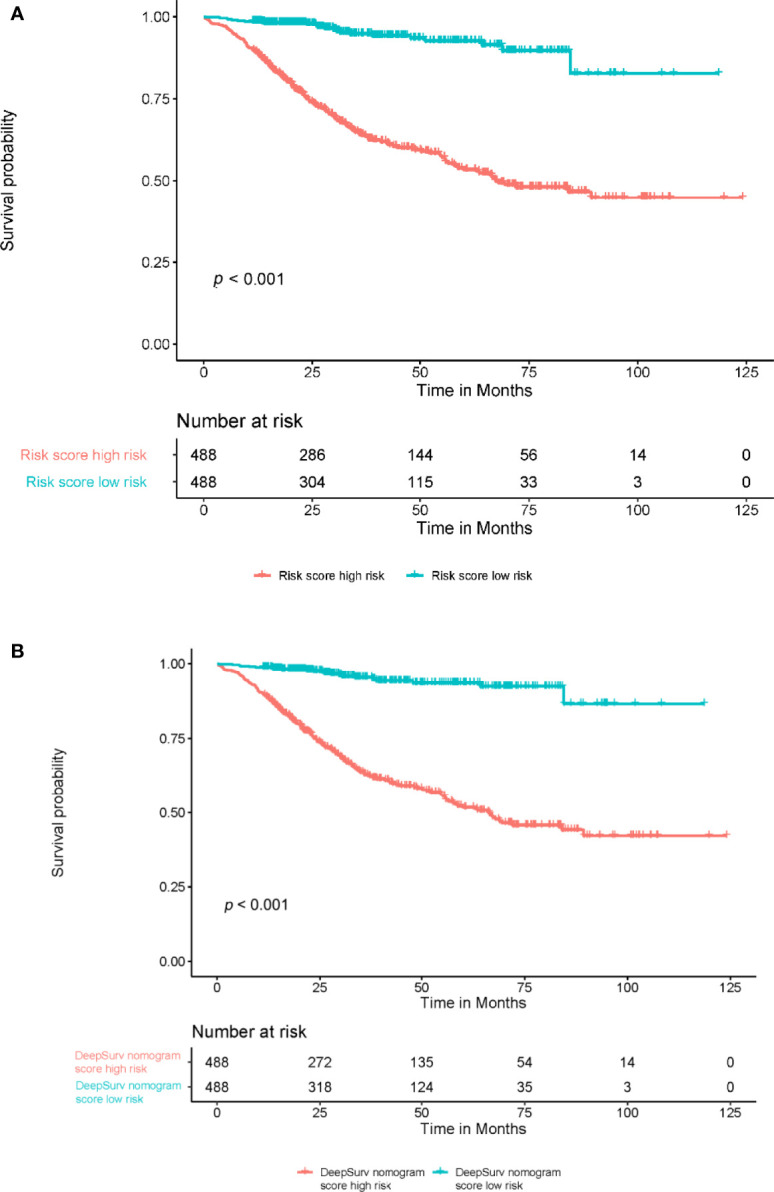
Predictive capacity of the risk score and the DeepSurv nomogram score. The Kaplan-Meier curve shows that both the risk score and DeepSurv nomogram score could effectively discriminate between patients with better and worse survival **(A, B)** (*P*<0.001, log-rank test).

The DeepSurv model, based on radiomics, yielded a C-index of 0.790 and 0.752 in the training and validation cohorts, respectively. Risk scores with independent risk factors were integrated to construct a clinical plus DeepSurv model (DeepSurv nomogram) using a multivariate CPH regression analysis. The C-index values of the clinical plus DeepSurv model were 0.821 and 0.768 in the training and validation cohorts, respectively. This model outperformed both the clinical and TNM models (C-index: 0.749 and 0.745 in the training and validation cohorts, respectively). The C-index of the multimodality prediction model is shown in **Table 3**. The DeepSurv nomogram for prediction performance of the 3 and 5 year survival was generated based on the risk score, age, TTF-1, Ki-67, and stage **(** **Figure 4****)**. Further, a calibration curve was drawn for these patients. The estimated versus observed values for 3 and 5 year survival probabilities intersected the 45° line, showing that the predicted probability was very close to the actual survival time of patients **(** **Figure 5****)**. In addition, the model showed a good calibration with *P*=0.39 in the GND test. Both the risk score and DeepSurv nomogram score demonstrated good risk stratification capacity in the Kaplan–Meier analysis of these patients **(** **Figures 6A, B****)**. The integrated Brier scores for the nomogram was 0.106 and 0.128 in the training and validation cohort, respectively, providing a more precise prognosis of OS than other models and systems **(** **Table 3****)**.

**Table 3:** Harrell’s concordance index of the different modalities.

Modalities	Training cohort (n = 542)		Validation cohort (n = 231)		*p*-value*
	C-index 95%CI	Brier Score	C-index 95%CI	Brier Score	
DeepSurv model	0.790 (0.757,0.823)	0.115	0.752 (0.671,0.832)	0.129	<0.001
Clinical model	0.788 (0.739,0.838)	0.119	0.739 (0.658,0.820)	0.137	<0.001
DeepSurv nomogram model	0.821(0.772,0.870)	0.106	0.768 (0.686,0.849)	0.128	
TNM model	0.749 (0.705,0.794)	0.128	0.745 (0.672,0.818)	0.116	0.002

*Compared to DeepSurv nomogram model.

C-index, Harrell’s concordance index.

### Clinical Use

**Figure 7:**
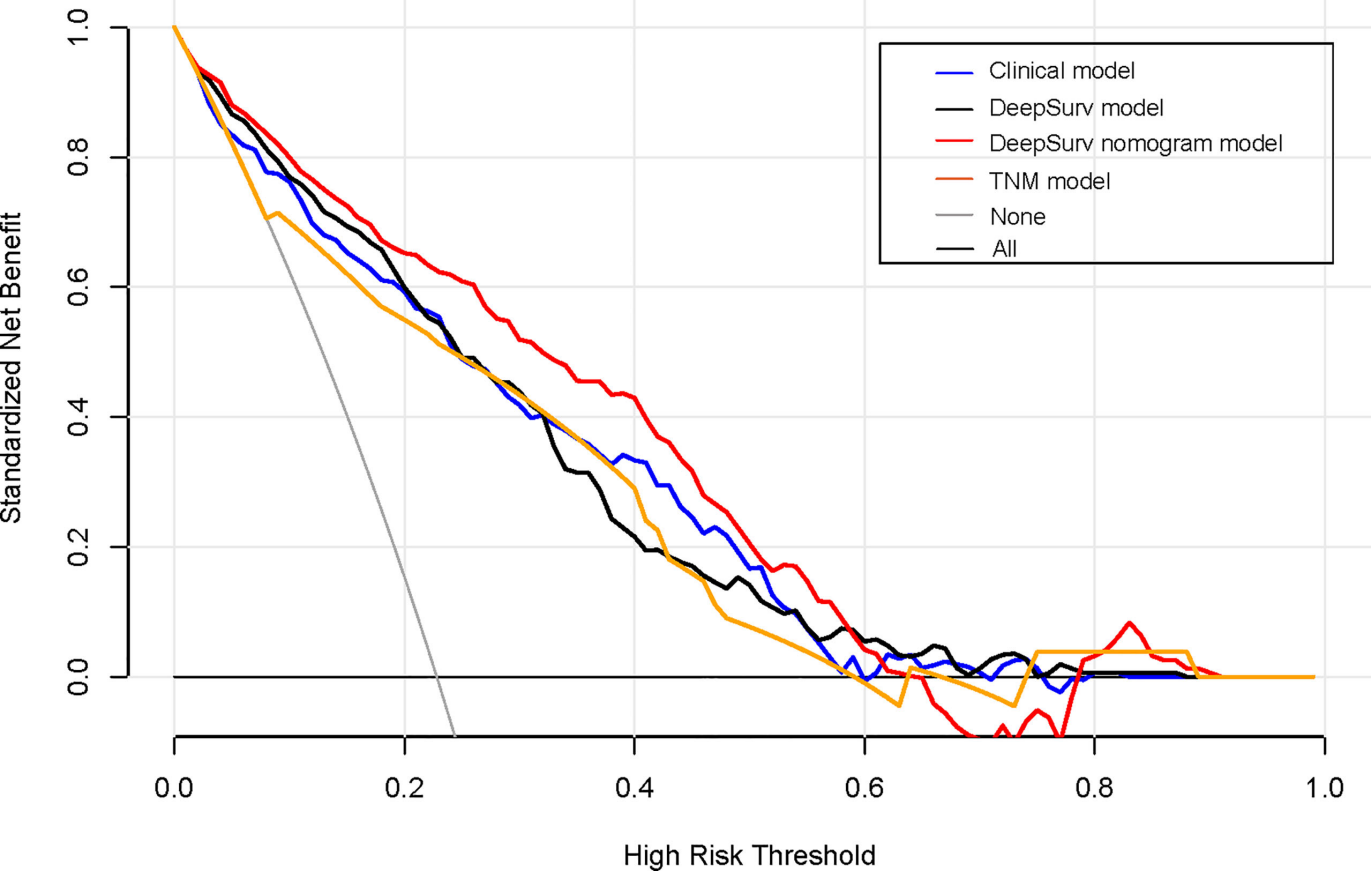
Decision curve analysis for each model. The y-axis measures the net benefit, which was calculated using true-positive and false-positive results. The clinical plus DeepSurv model had the highest net benefit at a threshold of 0.1 to 0.9 among all positive (line labeled “All”) and negative predictions (line labeled “None”), compared to the three other models (line labeled “clinical model, deep learning model, and TNM model”).

A decision curve analysis was performed to determine the clinical usefulness of the DeepSurv nomogram by quantifying the net benefits at different threshold probabilities. This showed that the DeepSurv nomogram had a higher overall net benefit as compared to other clinical models across the majority of reasonable threshold probabilities, as shown in **Figure 7**.

### Guide to Individualized Adjuvant Chemotherapy for Patients With NSCLC

**Figure 8:**
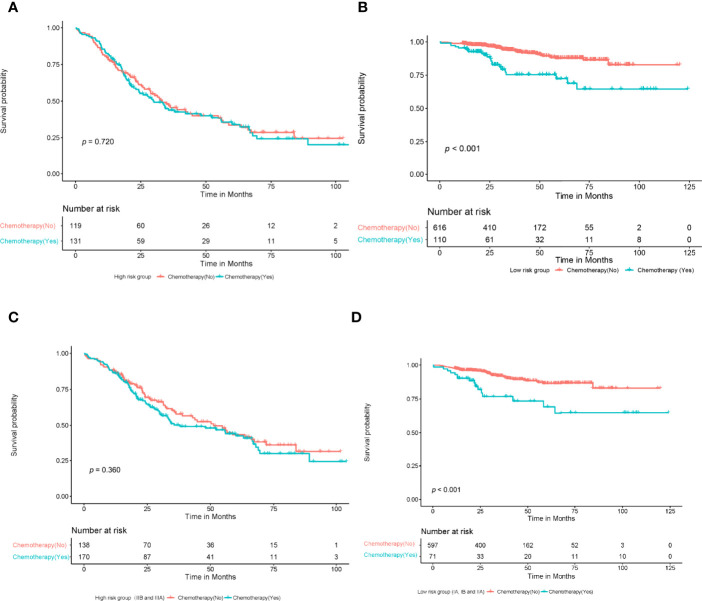
A Kaplan-meier analysis and log-rank test were performed to determine the survival rate of patients at high and low risk who with or without adjuvant chemotherapy. The results showed that there was no significant difference in survival rate among high-risk patients who with or without chemotherapy **(A)** (*P*=0.720, log-rank test); Patients in the low-risk group who received chemotherapy had a lower survival rate than those who did not **(B)** (*P*<0.001, log-rank test). There was no significant difference in survival rate among high-risk patients (IIB, IIIA) who with or without chemotherapy **(C)** (*P*=0.360, log-rank test); Patients in the low-risk group (IA,IB, IIA) who received chemotherapy had a lower survival rate than those who did not **(D)** (*P*<0.001, log-rank test).

The patients were divided into high- and low-risk groups according to the cutoff value of the DeepSurv nomogram score, and the sensitivity of patients to chemotherapy was analyzed. The results showed no statistically significant difference in the survival rate of patients in the high-risk group, irrespective of administration of adjuvant chemotherapy (*P*=0.720). In contrast, the prognosis of the low-risk group displayed a statistically significant difference, with a poorer prognosis in patients who had received chemotherapy (*P*<0.001). In addition, a Kaplan–Meier analysis and log-rank test were conducted on the survival rate of the high-risk group (IIB and IIIA), regardless of administration of adjuvant chemotherapy. This showed no statistically significant difference in the survival rate of the high-risk group, irrespective of whether they underwent adjuvant chemotherapy or not (*P*=0.360). In contrast, the prognosis of the low-risk group(I A、I B and II A) displayed a statistically significant difference, with a poorer prognosis in patients who had received chemotherapy (*P*<0.001; **Figures 8A–D**).

## Discussion

This study constructed and validated a DeepSurv nomogram based on CT radiomic features and independent risk factors. This DeepSurv model exhibited improved OS prediction performance in patients with NSCLC, compared with other models and systems with a C-index of 0.821 and 0.768 in the training and validation cohorts, respectively. It also exhibited good calibration evaluation and risk stratification capability. However, our results show that both high- and low-risk patients did not benefit from chemotherapy.

In recent years, artificial intelligence has been developing rapidly in the field of lung cancer. In our study, we used a new algorithm, DeepSurv, to construct the risk scores. Deep learning is useful for large-scale datasets. DeepSurv is a multi-layer perceptron similar to the Faraggi–Simon network (41, 42). However, it allows a deep architecture (i.e., more than one hidden layer) and applies novel deep learning techniques, such as weight decay regularization, rectified linear units, batch normalization, and dropout (28). Therefore, the DeepSurv model works like the standard linear CPH model, but out performs it in predicting survival data with linear and nonlinear risk functions. We built a DeepSurv nomogram with the highest C-index by combining the risk score and clinicopathological factors. In the clinical model, age (cutoff, 67) (HR, 1.039; 95% CI, 1.021–1.058), TTF-1 (positive) (HR, 0.623; 95% CI, 0.438–0.885), Ki-67 (high expression) (HR, 1.663; 95% CI, 1.191–2.322), and stage IIIA (HR, 8.731; 95% CI, 5.474–13.927) were the independent risk factors. These results were consistent with those of previous studies. TTF-1 is expressed in both the thyroid and lung tissues, and plays an important role in cell differentiation. The impact of TTF-1 on the prognosis of patients is still controversial; however, some studies have reported that TTF-1 positivity is better for the prognosis of patients (43, 44). Ki-67 is an important cell proliferation marker and is related to the prognostic value of some tumors. However, it has a negative effect on the prognosis of NSCLC (45, 46). By integrating these clinical factors and the DeepSurv algorithm’s output, our DeepSurv model demonstrated better performance in predicting the prognosis than that in previous studies. Yang et al. (47) developed a radiomic nomogram by combining the optimized radiomic signatures extracted from 2-D and/or 3-D CT images and clinical predictors, to assess the OS of patients with NSCLC. Their radiomics nomogram showed a significant improvement in patient survival prediction, with a C-index of 0.747, 0.729, and 0.710 in the training, internal validation, and external validation cohorts, respectively. Wang et al. (48) built a model integrating clinical, hematological, and CT radiomic features in predicting the survival of patients with locally advanced NSCLC. They found that the integrative nomogram achieved a C-index of 0.792 and retained 0.743 in the cross-validation analysis; thus, outperforming radiomic, clinical, or hematological models alone. Huang et al. (49) developed a radiomic signature to estimate disease-free survival (DFS) in patients with early-stage (stage I–II) NSCLC. They showed that the radiomic signature was significantly associated with DFS. Incorporating the radiomic signature into the radiomics-based nomogram resulted in better performance in the estimation of DFS (C-index: 0.72) than incorporating the radiomic signature into the clinicopathologic nomogram (C-index: 0.69). The neural network can also serve as a recommender system by including a categorical variable representing a treatment group. This can be used to provide personalized treatment recommendations based on an individual’s calculated risk. Our research shows that risk scores can stratify patients’ risk and provide clinical evidence for additional therapy or intensive follow-up for patients at high-risk or with a poor prognosis.

Furthermore, the calibration curve of the DeepSurv nomogram showed that the predicted survival time was close to the actual survival time. Our prediction model for prognosis displayed good stability and reliability. The decision curve analysis showed that the DeepSurv nomogram had a higher overall net benefit than three other clinical models across the majority of reasonable threshold probabilities. This shows that the risk score and DeepSurv nomogram have more potential in postoperative prognosis assessment. However, we believe that it is still necessary to conduct a large-scale independent prospective multicenter cohort to verify our results.

Finally, we aimed to use DeepSurv to guide individualized adjuvant chemotherapy for patients with NSCLC. No significant difference in the survival rates was observed in the high-risk group, irrespective of the use of adjuvant chemotherapy. In contrast, the prognosis of the low-risk group displayed a significant difference, with a poorer prognosis in patients who had received chemotherapy. This indicates that high-risk patients do not benefit from adjuvant chemotherapy. Additionally adjuvant chemotherapy alone did not improve the survival rate of high-risk patients with an advanced clinical stage, suggesting that the clinical stage may require additional treatment or a close follow-up. In addition, the low-risk groups did not appear to benefit from adjuvant chemotherapy, and it may lead to a poorer prognosis. Our research results are consistent with the extant literature (50). Based on the findings of our study, the prognosis of NSCLC can be predicted to analyze the independent risk factors that affect the prognosis of patients. Further, individualized treatment of patients can be guided.

Like most studies, our study has several limitations (51). First, this is a retrospective study, so there may be selection bias. Second, although the sample size is slightly larger than that in previous studies, it is a single-center study with no external verification. Third, genomic characteristics were not considered. The genetic phenotype of the tumor may explain the individual differences in survival prognosis at a biological level. We will integrate such data in future studies.

In conclusion, the DeepSurv nomogram based on the radiomic features and independent risk factor characteristics displayed a better prognostic and predictive performance for NSCLC. It can be used to guide the individualized treatment of high-risk patients. Therefore, DeepSurv nomogram can provide guidance to physicians in terms of personalized treatment recommendations.

## Data Availability Statement

Data available on request due to privacy/ethical restrictions.

## Ethics Statement

The Institutional Review Board of Affiliated Jinling Hospital, Medical School of Nanjing University approved this retrospective study and waived the need to obtain informed consent from the patients.

## Author Contributions

BY conceived the idea of the study. BY, CL, RW, JiaZ, AL, LM, JinZ, SY, LZ, and CZ collected the data. LZ and GL performed image analysis. BY wrote the manuscript. YG and XT performed the statistical analysis. YG, LZ, and GL edited and reviewed the manuscript. All the authors discussed the results and commented on the manuscript. All authors contributed to the article and approved the submitted version.

## Funding

This work was supported by the National Natural Science Foundation of China (NSFC) (82160348 for BY, 82127806 for GL).The program for Cultivating Reserve Talents in Medical Disciplines from the Health Committee of Yunnan Province (H-2018008 for BY). Special project of Organ Transplantation Clinical Medical Center in Yunnan Province (2021YZ-ZX-05 for BY). And National Key R&D Program of China(2020AAA0109505 for GL).

## Conflict of Interest

Authors YG and XT were employed by Siemens Healthineers Ltd.

The remaining authors declare that the research was conducted in the absence of any commercial or financial relationships that could be construed as a potential conflict of interest.

## Publisher’s Note

All claims expressed in this article are solely those of the authors and do not necessarily represent those of their affiliated organizations, or those of the publisher, the editors and the reviewers. Any product that may be evaluated in this article, or claim that may be made by its manufacturer, is not guaranteed or endorsed by the publisher.

## Abbreviations

CI, confidence interval; C-index, Harrell’s concordance index; CEA, carcinoembryonic antigen; HR, hazard ratio; LMR, Lymphocyte/monocyte ratio; NSCLC, non-small cell lung cancer; NOS, not otherwise specified; NLR, Neutrophil/lymphocyte ratio; OS, overall survival; PLR, Platelet/lymphocyte ratio; TNM, tumor-node-metastasis.
